# Fluxion Dilutions

**Published:** 1881-10

**Authors:** Wm. Jefferson Guernsey

**Affiliations:** 4430 Frankford Avenue; Philadelphia


### "FLUXION DILUTIONS."

WM. JEFFERSON GUERNSEY, M. D., PHILADELPHIA.

THAT Dr. Price has "the ability to perfectly express an idea,"* will not be here disputed, but if he imagines that his mere statement of "I don't believe" will be accepted for the proof he so much needs, he must indeed think his friend devoid of "the ability to understand."

If I *have* been "unjust," it is not that I have not read the original article carefully, nor that it has not received deserving thought. Would that such were the case.

Dr. Price takes exception to the application of the term "potency" to our higher attenuations. So be it; his remedies will not be as potent without more dilution. Hahnemann did indeed teach that there were three essentials in this potency question, viz.: dilution of liquids, trituration of insolubles and succussion. But he does *not* give, as a reason for using succussion, that ridiculous notion, which Dr. P. quotes from Dr. S. A. Jones, that "it is necessary to the subdivision of matter," and who further states that "a moderately high fluxion preparation does actually contain as much drug substance as a very low Hahnemannian preparation," and that the reported cures

---

*See August number of this journal, page 366.

by some are really due to "infinitesimal particles of drug substance that happen not to have been washed out of the apparatus." I will let Hahnemann answer in his own words:* "A solution of salt, or bitter substance, becomes less salt or bitter in proportion as water is added to it, until it finally loses its taste altogether, *no matter how much we shake it;* in a similar manner a colored solution finally loses its color altogether by sufficient quantity of water being added to it." And further, he declares the necessity of dilution by saying that "dilution cannot be accomplished by simply triturating or shaking the original substance, were we to do it for ever so long a period."

It is with pleasure that the writer can inform Dr. P. that the fluxion dilution process of Swan *begins* with a *one thousand*, or higher potency.† And Dr. Skinner says that "many of his (Swan's) potencies were grafted on Dunham's 200th, which were all hand-made."‡

Fincke's patent states that "fluxion commonly starts from at least the third Hahnemannian potency, if not, the substance is entirely soluble in water, as *Nat. mur.*" Let me now ask, how much "drug substance" we have to start with? Again, as to the possibility of making a high potency (dilution, if you please), let me quote from Dr. Skinner again:‖ "After great labor, we have arrived at the following approximate values between Fincke's and Hahnemann's potencies, and we are quite prepared not only to stand by them, but to demonstrate their truth beyond all doubt:

| | | | | | |
|---|---|---|---|---|---|
| "Fincke's | 1 M. | is approximately | Hahnemann's | 151 | cent. |
| " | 10 M. | " | " | 1,506 | " |
| " | CM. | " | " | 15,053 | " |
| " | MM. | " | " | 150,530 | " " |

All this is verified on pages 324, 325 and 326.

Again,§ Dr. Skinner asserts: "Hahnemann's 150 M. *can* be made, and *has* been made by myself many a time in 11h., 6′, 40″, "by the fluxion process of Fincke;" and further on, that "Hahnemann's MM. can be made by his 'fluxion attenuator' in 3d., 2h., 4½m." All this is, of course, without succussion. My friend has, however, preferred the word "friction." Accepting his choice, let me inquire if there be no "friction" where water is forced up into the glass and flows out at the top? or where the reverse plan is used?

Once more. I take exception to his admission, "*some* of these

*Preface to "Chronic Diseases," Vol. V.

†"*The Organon*," Vol. II. p. 398.

‡"*The Organon*," Vol. III, p. 193.

‖"*The Organon*," Vol. III, p. 323.

§"*The Organon*," Vol. III, p. 327.

preparations *may* act." He here tells the truth, but not the whole truth, and he infers what is something but the truth. *All* of them act. And they *do* act. A strange coincidence, that of all my high dilutions, a little "drug substance" should have remained in each.

Hahnemann's principle was "to give each patient" *just enough* medicine to effect the cure. Could we but imitate the grocer who, to obtain the desired weight of sugar, throws more than enough into the scales, and then scoops out the surplus, we could medicate *ad libitum*. But no, the proper method (and if my friend thinks this madness, "yet there is method in it") is to give a patient but *one dose* when beginning a case or changing to a new remedy. This must not be interfered with. It should be allowed to act until we are *sure* of one of two things, viz.: that it is the wrong medicine; or, that it is the right medicine, and that he needs more of it. If no improvement takes place in a reasonable time, which our judgment must dictate (from one hour to a week, usually twenty-four hours in acute troubles), we may conclude that the former is the case, and act accordingly. If we are favored with an improved condition, which gradually becomes beautifully less, we give three, four, six or eight powders of the same remedy at reasonably short intervals, and wait correspondingly long for an improvement, which will almost surely follow. *I, invariably*, do without medication so long as improvement continues. One point should always be borne in mind in reconsidering an unsuccessful prescription: is the remedy last given thoroughly indicated? Unless an *unquestionable* answer can be given to this self-interrogation, the case must receive *Sac. lac.* until the office and library are reached. If the remedy was a poor choice, make a better one. If you are *sure* you are right, give more of it, as suggested above. Give us such care and the use of *high* attenuations, and the "jack and the game" will be ours. He is welcome to the "*low*."

Before closing, I cannot refrain from reference to his reported cures which he prefaces with the remark that "even the greatest fluxion enthusiast *cannot* excel," etc. Case I, of Miss L. (neuralgia) was one indicating *Pulsatilla*, no doubt. His potency cured "in *a week*." Whether his potency did not contain a sufficiency of "drug" particles, we know not, but if Dr. P. will accept it, he is welcome to a graft from Fincke's 52 M, which I think would have declared peace in a few hours, or less. Case II, a diarrhœa of "some *hours'* standing," cured in *twelve hours*. The word "standing" should be here omitted, and one dose and one stool would have ended the trouble.

Case III, B. F. S. (one of acne), cured under a few changes of medicine in *six months.* Often a stubborn affection, but six months the maximum of duration. Cases IV and V are exceptions to his rule.

And now may the saints defend us from any further "succussion" of this letter. Dr. Price is understood, and the writer has given his best expression to his poor idea. "May he rest in peace."

4430 Frankford Avenue, *August* 31*st.*

